# Perceptions of Global Health Engagements in Relation to the COVID-19 Pandemic Among Health Care Workers and Administrators in Western Kenya: Protocol for a Multistage Qualitative Study

**DOI:** 10.2196/41836

**Published:** 2023-07-19

**Authors:** Erick Amick, Violet Naanyu, Sherri Bucher, Beverly W Henry

**Affiliations:** 1 College of Health and Human Sciences Northern Illinois University DeKalb, IL United States; 2 Susan and Richard Kiphart Center for Global Health and Social Development Crown Family School of Social Work, Policy, and Practice University of Chicago Chicago, IL United States; 3 School of Arts and Social Sciences Moi University Eldoret Kenya; 4 Department of Pediatrics School of Medicine Indiana University Indianapolis, IN United States

**Keywords:** global health, global health engagements, GHEs, health care workers, local perspectives, low- and middle-income countries, LMICs

## Abstract

**Background:**

There has been significant interest in global health in low- and middle-income countries (LMICs) among individuals living in high-income countries (HICs) over the past 30 years. Much of the literature on global health engagements (GHEs) has been presented from the perspective of individuals from high-income countries. Local stakeholders such as health care workers and health care administrators represent critical constituencies for global health activities, yet their perspectives are underrepresented in the literature. The purpose of this study is to examine the experiences of local health care workers and administrators with GHEs in Kenya. We will explore the perceived role GHEs play in preparing the health system to address a public health crisis, as well as their role in pandemic recovery and its aftermath.

**Objective:**

The aims of this study are to (1) examine how Kenyan health care workers and administrators interpret experiences with GHEs as having advantaged or hindered them and the local health system to provide care during an acute public health crisis and (2) to explore recommendations to reimagine GHEs in a postpandemic Kenya.

**Methods:**

This study will be conducted at a large teaching and referral hospital in western Kenya with a long history of hosting GHEs in support of its tripartite mission of providing care, training, and research. This qualitative study will be conducted in 3 phases. In phase 1, in-depth interviews will be conducted to capture participants’ lived experience in relation to their unique understandings of the pandemic, GHEs, and the local health system. In phase 2, group discussions using nominal group techniques will be conducted to determine potential priority areas to reimagine future GHEs. In phase 3, in-depth interviews will be conducted to explore these priority areas in greater detail to explore recommendations for potential strategies, policies, and other actions that might be used to achieve the priorities determined to be of highest importance.

**Results:**

The study activities commenced in late summer 2022, with findings to be published in 2023. It is anticipated that the findings from this study will provide insight into the role GHEs play in a local health system in Kenya and provide critical stakeholder and partner input from persons hitherto ignored in the design, implementation, and management of GHEs.

**Conclusions:**

This qualitative study will examine the perspectives of GHEs in relation to the COVID-19 pandemic among Kenyan health care workers and health care administrators in western Kenya using a multistage protocol. Using a combination of in-depth interviews and nominal group techniques, this study aims to shed light on the roles global health activities are perceived to play in preparing health care professionals and the health system to address an acute public health crisis.

**International Registered Report Identifier (IRRID):**

PRR1-10.2196/41836

## Introduction

### Background

Over the past 30 years, there has been growing interest in global health volunteering and service-learning trips in low- and middle-income countries (LMICs) among individuals living in high-income countries (HICs) [1,2]. This is reflected in a large increase in the number of colleges, universities, and medical schools that offer programs of study and experiences in global health to their students. Across the United States, educational institutions of all sizes have begun offering global health programs to undergraduates and graduate students [3,4]. The desire for global health experiences is also observed among American medical students, 31% of whom reported having a global health experience in medical school in 2015, up from just 15% in 1998 [5].

Global health activities taking place in LMICs vary widely, with short-term clinical services being among the most commonly described in the literature, particularly among medical trainees [6,7]. The terminology used to describe these programs also varies considerably, with “international medical electives,” “short-term medical missions,” and “short-term experiences in global health” being just a few terms used to describe these activities [8-13]. The term “global health engagements” (GHEs) perhaps describes these activities in more comprehensive and inclusive terms and provides consideration to the fact that interest in global health has not been limited to the boundaries of higher education, or to those with specialized clinical skills. Religious organizations, civic clubs, nongovernmental organizations, corporate groups, and even for-profit volunteer placement companies have offered GHEs for those without specialized skillsets to participate in global health volunteering abroad [14]. As such, opportunities abound for both skilled health professionals and nonskilled lay people to travel to LMICs to participate in global health activities, including medical missions, service-learning projects, and a wide range of volunteer service activities [15,16]. Ranging from a few days to several weeks, GHEs take place in LMICs around the world with varying degrees of coordination with local stakeholders [7,11,14]. A core challenge of GHEs is that traveling volunteers often lack adequate training and preparation to work in the environment, regardless of their educational background or prior training, and are frequently sent into LMIC settings without formal needs assessments having been conducted nor adequate consultation with in-country stakeholders regarding local needs. This implies that many global health interventions may be subjective solutions posed by HIC participants rather than bidirectional efforts to address local needs and priorities [17-20].

GHEs are big business. Fueled by a growing awareness of global issues and participant motivations of “giving back” and “doing good,” annual expenditures on GHEs have been estimated to be well in excess of US $3 billion from the United States alone [10]. A significantly increased interest in GHEs in recent years has prompted increased scientific inquiry and academic analysis on the subject. GHEs have been generally portrayed as providing value to the health care systems of LMICs, although objective assessments of these activities are infrequent [11]. A common critique of GHEs focuses on ethical concerns, in that they might allow an opportunity for students and nonskilled volunteers to “practice on the poor” by providing care beyond their skillset [21]. Recently, experienced global health practitioners have sought to bring greater awareness of this and other ethical concerns and have called for the development of guidelines for ethics training, predeparture preparation, and management of participants to improve the otherwise largely unregulated activities in GHEs [4,11,22,23]. Recent critiques of these questionable practices have also brought attention to inequities in global health activities. It has been argued that GHEs perpetuate colonial mindsets and power asymmetries between HIC and LMIC individuals and organizations [24,25]. In recent years, there has been growing awareness to examine these inequities in an effort to “decolonize” global health practices in favor of more equitable practices, including greater decision-making regarding the management of GHEs. Despite increased interest to critically evaluate global health practices through the lens of decoloniality, much of the scientific literature on global health efforts and GHEs has been presented in overwhelmingly positive terms, and largely from the perspective of HIC participants [11,14].

Examples of recent studies focused on HIC individuals participating in GHEs include those examining satisfaction and motivations for participation among volunteers; the impact on long-term career paths for students; and cultural competency skills development [26-29]. In addition to allowing HIC individuals to build their resumes, engagement in GHEs provides participants (particularly medical trainees) the ability to see tropical diseases firsthand or to develop cross-cultural communications skills in an effort to promote themselves as well-rounded and experienced candidates in the job market [4,15]. HIC participation in GHEs has also been shown to influence career choices, leading participants to choose careers in public service [30-32]. Although examining GHEs from the perspective of HIC participants is important, these individuals make up only a small portion of the total number of stakeholders involved with global health activities occurring in LMICs. Local stakeholders, such as health care workers and health care administrators, represent a critical component of GHE activities, yet research focused on their perspectives is far less common. A recent literature review revealed only 31 studies reporting local stakeholder perspectives from LMICs, mostly consisting of multicountry surveys from LMICs around the world, although none focused solely on Kenya, despite the regular occurrence of GHEs in this setting. Studies of GHEs have shown that LMICs are often recipients of GHEs due in part to longstanding social, cultural, and political ties between institutions and communities within the host country and the sending country [6,14,15]. Since 1964, Kenya has maintained favorable and consistent diplomatic relations with the United States following its independence from Britain in December 1963 [33]. This long history has no doubt contributed, at least in part, to many Americans’ perception of Kenya as a safe and stable country—a factor also known to attract GHEs [14]. Kenya’s status as an English-speaking country and a top-notch safari destination are possible considerations that make Kenya an attractive location for some individuals pursuing GHEs, while others may be drawn to global health “hotspots” on the African continent due to (perhaps misguided) perceptions of needs related to poverty or health crises [15]. Whatever the motivations, Americans and other HIC individuals pursuing GHEs have maintained a notable and sustained presence in Kenya in recent decades.

### COVID-19 Global Pandemic

The advent of the COVID-19 global pandemic significantly disrupted day-to-day activities around the world, including global health activities in Kenya [34]. This unprecedented shift has impacted local health care workers, health care administrators, and the health systems in which they work. Like in many other countries, health care workers in Kenya have experienced increased stressors, anxiety, insomnia, depression, and burnout [35-37]. Although occupational challenges are being increasingly well documented, far less is known about the potential new understandings health care workers and health care administrators are gaining as they make meaning of their work, the health care system, and the role of global health activities in relation to the COVID-19 pandemic in Kenya. We developed our study protocol to leverage the unique and unprecedented opportunity to explore how health care workers and health care administrators understand their new reality working in an environment where GHEs, which were previously ubiquitous, have greatly declined over a significant period of time. In this study, we will ask participants to reflect on their history with GHEs and consider how prior experience with these activities may have shaped them professionally, and the health system broadly, to provide care in the face of the COVID-19 pandemic. Findings from this study may contribute to the field of global health research by providing a more nuanced understanding of the roles—expected or actual—GHEs play in the professional lives of those working within LMIC health systems, as well as their perceptions of the functioning of the system itself. The paucity of current research on local LMIC stakeholder perceptions of GHEs means that far less is known about how individuals make meaning of these activities in comparison to their HIC colleagues. Understanding the local perspectives of GHEs and global health inequities is of increased importance in the era of the COVID-19 pandemic. Further research to examine GHEs from the local perspective is necessary to paint a more complete picture of how these activities are perceived by those who host and manage global health activities in Kenya, particularly in the context of the pandemic.

### Study Aims and Objectives

The purpose of this study is to examine local health care workers’ and administrators’ experiences with GHEs in Kenya and the role they are perceived to play in preparing individuals and the health system to address the COVID-19 public health crisis and their unfolding role in pandemic recovery and its aftermath. The primary aim of this study is to examine how Kenyan health care workers and administrators interpret experiences with GHEs as having advantaged or hindered them and the local health system to provide care during the COVID-19 pandemic.

Our study protocol is guided by the following research questions: (1) In what ways have GHEs affected health care workers’ and administrators’ professional lives and their perceptions of the local health system? (2) How have previous experiences hosting GHEs positioned these individuals and the health system to deliver care during the COVID-19 crisis? and (3) What are stakeholder priorities with regard to the design, implementation, and management of GHEs in Kenya in the future?

To achieve the primary aim of our study, one-on-one in-depth interviews will be conducted among health care workers and administrators in the fields of pediatrics, adult medicine, and mental health care. Interviews will explore how participants make meaning of prior experiences of GHEs in relation to their work in a health system under the stress of a public health crisis. Their reflections may reveal new insight into the role GHEs may play in strengthening the capacity of the health care system as well as those who work within it.

A secondary aim of this study is to identify stakeholder priorities and explore recommendations for future GHEs to achieve maximum benefit in the health care system after the COVID-19 pandemic. We will achieve this aim by using a group consultation process, nominal group techniques (NGTs), followed by one-on-one in-depth interviews. NGTs use a highly prescriptive, multistep process that engages each group participant individually and gives equal voice across the group, thereby minimizing opportunities for stronger personalities to dominate conversations and influence group dynamics. NGTs were chosen in lieu of focus group discussions for several reasons. Primarily, NGTs offer a high level of efficiency in achieving group consensus in comparison to focus groups, which can be significantly impacted by group dynamics, dominant personalities, and biases of participants and researchers [38]. Equally important in the selection of NGTs is their proven use in research in health care research, including among individuals working in health care in the African context [39-43]. In our study, NGTs will be used to generate a prioritized list of recommendations among health care workers and administrators, which will be examined further in subsequent in-depth interviews. In-depth interviews will identify potential strategies to implement recommendations generated during NGT activities. Together, these methods will result in stakeholder-identified priorities as well as actionable strategies necessary to implement recommended innovations for future GHEs in Kenya.

## Methods

### Study Design

Qualitative methods are particularly well-suited to understanding disruptive social events, including disasters and crises, to explore meaning-making and the experiences of those living through these events [44]. Using 2 qualitative methods, our study will explore the perceptions of GHEs among individuals and groups from 2 key stakeholder groups who were particularly impacted by the COVID-19 public health crisis: health care workers and health care administrators. Conducted in 3 phases, our study will ask participants to reflect on their experiences working in the health care system prior to and during the COVID-19 pandemic to examine how their understandings of GHEs have shifted. Participants will be asked to consider how the presence of GHEs or lack thereof may have benefited, or disadvantaged, them to provide care during an acute public health crisis. Our research will also explore participants’ opinions, views, and perspectives on the essential elements of GHEs that are beneficial, as well as identify the aspects of these activities in which innovations can be made to improve their design, implementation, and management in the future.

Using in-depth interviews, phase 1 of the study will explore perceptions of, and experiences with, GHEs among frontline health care workers (eg, nurses and physicians) and health care administrators (eg, individuals responsible for decision-making and the day-to-day management of health care facilities). The research team will use NGTs in phase 2 to develop a stakeholder-prioritized list of actions and recommendations that can be used in future GHEs in Kenya. Similar to focus groups, NGTs are used to gather input from stakeholder groups regarding a specific topic of interest. Unlike focus groups, which rely on group discussions guided by a facilitator, NGTs use a highly structured protocol where participants are individually asked to generate ideas, discuss them as a group, and rank or score those ideas. This results in building consensus on the discussion topics, which can be used to determine priorities for action to solve problems. This is a useful method to compare the viewpoints and priorities of stakeholder groups. Two NGTs will be conducted in this study, 1 for each of the 2 stakeholder groups (ie, health care workers and health care administrators). Phase 3 will use another set of in-depth interviews to further explore potential recommendations identified in phase 2 regarding the design, implementation, and management of GHEs in pandemic recovery and beyond. The findings will offer insight from the local perspective regarding the role GHEs play in local health systems in Kenya and provide critical stakeholder input on the management of future GHEs.

### Study Setting

Participants will be recruited from the flagship hospital of a network of health care facilities, which regularly hosts a wide variety of global health activities, headquartered in the Kenyan city of Eldoret, in Uasin Gishu county [45,46]. The network of health care facilities began with a single facility through a partnership of universities in Kenya and the United States. Established in the early 1990s in response to the HIV/AIDS crisis in Kenya, it now comprises a network of more than 800 care facilities across western Kenya. Today, this system provides primary and specialized health care to more than 8 million people, including more than 150,000 in its flagship HIV/AIDS program, and pursues a tripartite mission of providing health care and training and conducting research in partnership with many universities across North America [47]. For the past 30 years, this health system has had a consistent flow of North American faculty, researchers, clinicians, students, and affiliated personnel to participate in global health service and learning opportunities in the fields of medicine, pharmacy, dentistry, nursing, child health, and myriad other health professions. The constant presence of these HIC individuals over the past 3 decades makes this setting ideal to study local perspectives on GHEs and perceived impact of the COVID-19 pandemic on the health system.

### Ethics Approval

The research procedures presented in this study protocol have been approved by the Northern Illinois University Institutional Review Board (HS21-0286) and Moi University’s Institutional Review and Ethics Committee (IREC059/2021). Protocols were also reviewed by the study site’s research working groups, which are responsible for vetting research conducted at the network’s facilities prior to ethics reviews. Protocols have been approved by the behavioral and social science and public health and primary care working groups for primary and secondary review, respectively. A national research permit was also secured from Kenya’s National Commission for Science, Technology, and Innovation (NACOSTI/P22/16944).

### Study Participants

Health care workers and health care administrators were selected as the 2 primary categories of stakeholders of interest for this study because they offer 2 very important, yet potentially different perspectives, on GHE activities at health care facilities in western Kenya. This cross-sectional approach has been commonly used in health science research in African settings, including Kenya, and was chosen because it creates opportunities for a more comprehensive exploration of a broad range of perspectives than otherwise possible from a singular cadre of stakeholders [48,49]. First, as decision-makers and managers, health care administrators stand to offer viewpoints rooted in leadership and organizational perspectives, particularly in relation to carrying out activities and achieving the health care network’s tripartite mission. Their “30,000-foot view” can provide insight into the design and implementation of GHEs as well as the “how and why” decisions are made regarding them. In contrast, health care workers and other frontline staff offer different, much more granular perspectives of GHEs and can provide an up close and personal look at these activities. Frequently working alongside individuals pursuing GHEs, individuals in this stakeholder category have firsthand knowledge of the challenges and opportunities created by these activities and understand how they influence clinical environments for both patients and local providers. Collectively, both of these stakeholder groups offer an opportunity to explore perceptions on GHEs in Kenya during the COVID-19 pandemic.

We will recruit participants aged 18 years or older who are proficient in English, have worked at target facilities for a minimum of 2 years, and have either direct interaction with global health activities or have firsthand knowledge of their impact on their clinical facility and wider network’s system. Inclusion criteria include being a health care worker (eg, nurse, physician, and other clinical staff) or a health care administrator (eg, “in-charge” staff, facility leadership and management, other administrative staff) working in facilities providing pediatric, adult medicine, or mental health care services. Exclusion criteria include those aged younger than 18 years; those without the ability to speak English; individuals working at the target health facilities for less than 2 years; those without direct experience with, or knowledge of, global health activities in the facility; individuals who do not fall into the 2 stakeholder categories (ie, health care workers and health care administrators); and those working outside of pediatrics, adult medicine, and mental health care services.

### Recruitment Procedures

To elicit a range of perspectives, participants will be recruited from among individuals working in the fields of pediatrics, adult medicine, and mental health care. Recruitment flyers will be posted in staff areas of targeted facilities and at selected staff gatherings (eg, staff meetings), where members of the research team will briefly share information on the purpose of the study and provide contact details. Interested parties will be invited to contact members of the research team to arrange eligibility screenings and to discuss study activities. Once eligibility has been established, potential participants can be recruited into 1 or more phases of the study based on the individual’s interest and the study’s ongoing recruitment needs. Simultaneous recruitment for all 3 study phases is intended to provide greater flexibility, improve efficiencies, and counterbalance potential challenges due to the attrition of participants over the course of study activities. Purposive sampling will be used to achieve a balanced range of perspectives of participants across stakeholder categories as well as gender [50]. As such, male and female participants will be recruited into the study in roughly equal numbers into each of the 2 stakeholder categories (ie, health care administrators and health care workers).

**Figure 1:**
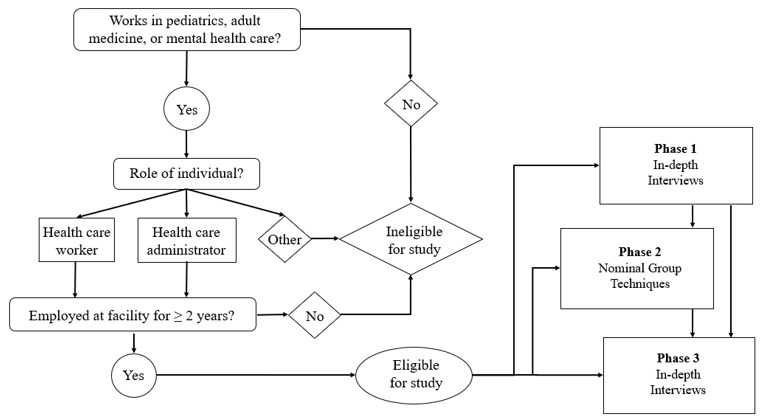
Recruitment eligibility decision tree.

Figure 1 shows the recruitment eligibility decision tree for basic recruitment and eligibility guidelines for study participation.

### Informed Consent

All research activities, including the informed consent process, will take place in a private room and informed consent will be obtained prior to eligible individuals’ engagement in any study activity. A member of the research team will provide eligible individuals with an overview of the nature of the study including that their participation is voluntary. Potential participants will be provided with all pertinent information including the study’s purpose, procedures, risks, and benefits. A member of the research team will explain how confidentiality will be maintained, including the management and storage of data for future use. The individual will be provided with information on their rights as a study participant, including that their participation in the research study is entirely voluntary and that participation can be withdrawn at any point during study activities.

After being given the overview of the study and their rights as a participant, the individual will be given ample opportunity to ask questions and to seek clarification on any points that require additional information. The potential participant will then be assessed to determine whether they understand the study. The research team member will assess the individual’s understanding of the goals of the study by asking “Can you tell me what this study is about?” The individual will then be asked (1) to name things they will be expected to do during the study; (2) to explain what they would do if they no longer wished to participate in the study; and (3) to identify potential risks and benefits of participating in the study. Respondents will be enrolled only if they are able to provide clear and correct answers to each item.

The individual will then be given a copy of the informed consent document to read in full. After the person is done reading the document, they will be asked if they understand the form and will be given the opportunity to ask clarification questions. After satisfying any remaining questions, the individual will be asked if they would like to participate in the study. The individual will be enrolled in the study by signing, dating, and returning the informed consent document to the research team member. The participant will be given the option to have a copy of the informed consent form to keep for their own records.

### Compensation

Eligible Kenyan health care workers and health care administrators will receive compensation for their time. Participants will receive 550 Kenyan shillings (approximately US $5) for each study component (ie, interviews and NGTs) in which they participate. Cash payments will be made at the completion of each interview or group activity.

### Data Collection Procedures

#### Overview

All data collection activities will be administered by a team of trained Kenyan research assistants experienced in qualitative research methods. All one-on-one interviews and NGTs will be audio-recorded and transcribed verbatim. Transcripts will then be verified and deidentified by members of the research team. All study records will be assigned a unique identification number and will be uploaded and stored in password-protected files on a cloud-based encrypted file storage service.

#### Phase 1

One-on-one in-depth interviews will be conducted to capture participants’ lived experience in relation to their unique histories and perspectives regarding GHEs and the local health system. Interviews will be conducted by Kenyan research assistants in order to facilitate an environment conducive to elucidating candid responses from participants. This is of particular importance given that previous research on stakeholder perspectives on GHEs suggests that participants are reluctant to make overly critical statements, particularly among HIC partners, for fear of losing access to resources and opportunities [51,52].

In-depth interviews will be conducted for the 2 stakeholder categories of interest: health care workers (target sample size: n=5) and health care administrators (target sample size: n=5). All in-depth interviews will be conducted in a private setting within the research areas of the study site, which is a teaching and referral hospital. In-depth interviews will be audio-recorded and will last approximately 1 hour. Interviews will conclude when questions or talking points (as outlined in research protocols) have been exhausted. Textbox 1 below outlines sample interview questions. Participants will be queried about their past experiences with GHEs and will be asked to provide their perspectives (beliefs, opinions, and viewpoints) on GHEs in Kenya. Participants will also be asked to reflect on how these activities have professionally shaped them, as well as the health system, to provide care during the COVID-19 pandemic (Textbox 1).

**Phase 1: sample interview questions.**

- What have you learned about the Kenyan health system during the COVID-19 pandemic?
  Probe: Strengths?
  Probe: Weaknesses?
- In your view, how has the prior history of global health activities shaped the local health system to face events such as the COVID-19 pandemic?
  Probe: Prepared the health system?
  Probe: Hindered the health system?
- How has your own prior history with global health activities shaped you professionally to face events such as the COVID-19 pandemic?
  Probe: Prepared you?
  Probe: Hindered you?

#### Phase 2

Group discussions using NGTs will be conducted in phase 2 of the study. NGTs are administered through a structured, multistep process in which participants are asked to (1) generate ideas, (2) share ideas in round-robin format, (3) clarify ideas presented, and (4) rank the ideas presented. This consensus-building research method is used to determine stakeholder priorities for action to solve problems among communities of interest. NGTs are similar to focus group discussions; however, they have unique strengths, namely the ability to equalize groups of participants and minimize opportunities for individual participants from dominating or influencing the group dynamic. Using the round-robin format, NGT facilitators engage each participant to speak one by one, creating an equal voice across the group and minimizing opportunities for stronger personalities to dominate the conversation [53]. Additionally, NGTs are noted for their usability and outputs, typically prioritized lists of actions, and can be a useful method to compare viewpoints and priorities of stakeholder groups [39]. NGT methods have also been shown to reduce researcher bias and have been applied frequently in health research, particularly among health care workers—including in sub-Saharan Africa [39-43,54].

A total of 2 NGTs will be conducted, 1 for health care workers (target sample size: n=8) and 1 for health care administrators (target sample size: n=8). Each NGT is anticipated to last between 2 and 3 hours and will be audio-recorded. NGTs will conclude after completing the steps of nominal group activities. The 8 steps outlined below describe the NGT process developed for this study:

1. Explain the process for NGTs: In the first step, facilitators will outlay expectations and answer questions participants may have regarding the process.
2. Silent idea generation: Participants will be asked to recall their past experiences with global health activities prior to the COVID-19 pandemic to reflect on aspects (eg, characteristics, policies, and management) to recall how these activities impacted them and their work, both positively and negatively. Participants will then be presented with the following prompt: “What changes do you feel are necessary for global health activities after the COVID-19 pandemic is over?” and will be asked to silently and independently generate ideas for 5 minutes.
3. Round-robin idea sharing: Participants share their ideas one at a time, 1 participant at a time. Participants may think of new ideas during this phase but can only share them at their designated turn. Ideas are recorded on a flip chart until no new ideas are uncovered.
4. Clarification: A brief discussion of ideas may follow for clarity of purpose for the ideas presented. Participants may ask questions of facilitators and other participants to clarify the ideas presented. Similar or overlapping ideas are grouped together and are discussed until all participants understand each concept presented.
5. Idea ranking: Participants are then asked to consider all the ideas presented and to vote in rank order based on their subjective determinations of importance. Participants are given a piece of paper to anonymously assign scores to the top 5 ideas according to their preferences. Scores will then be tallied by the facilitators and presented to the group for discussion. The number of ideas to be discussed will vary depending on how many ideas were generated in the second and third stages.
6. Discussion: Tallied scores are then presented to the group for a detailed discussion regarding the results. Participants will be invited to share their thoughts on the results. Participants can provide additional context to why certain ideas may have received higher scores and may ask questions of one another for clarification and to foster robust discussion. No new ideas will be introduced or discussed at this stage.
7. Reranking: After discussing results, participants will be asked to anonymously rank the ideas once more based on the discussion of the results from ranking in step 5. Participants will again assign scores to their top 5 ideas according to their preference in light of the recent discussion. Participants may remain consistent with their ranking selections or can change their selections according to changes in their opinions based on the discussion.
8. Sharing of results: Final scores will be tallied and presented to the group. The top-scoring ideas will be used as themes to explore in more detail through one-on-one in-depth interviews in phase 3.

Conventionally, NGTs are drawn to a close after a single round of ranking or voting and scores have been tallied (corresponding to step 5 in this study) [39]. Our study adds additional steps to include participants in a peer-to-peer discussion to further explore potential motivations behind their selections. The additional step for discussion (step 6) was added to this study to allow participants to engage with one another in dialogue similar to that of focus groups to share greater detail regarding their preferences for certain ideas over others. A second round of ranking (step 7) will provide participants with the opportunity to reassess their individual priorities in consultation with their peers. Collectively, these additional steps are intended to gather robust data to provide further insight into the groups’ interests in prioritizing key areas of action in relation to GHEs.

#### Phase 3

One-on-one in-depth interviews will be conducted based on the ranked priorities identified in phase 2. A semistructured interview guide will be used to further explore the priority areas identified in NGTs. These in-depth interviews will serve as a means to closely examine the similarities and differences of ranked priorities between health care workers and health care administrators, as well as to explore the contextual nuances of NGT discussions. Phase 3 participants will be recruited into groups of key informants, health care workers (target sample size: n=5), and health care administrators (target sample size: n=5). All in-depth interviews will be conducted in a private setting, will be audio-recorded, and last approximately 1 hour. Interviews will conclude when questions and talking points have been exhausted. Participants will be asked to share their perspectives on potential innovations identified in phase 2 regarding the implementation and management of global health activities in the future. Data collected in phase 3 will provide insight into recommendations for potential strategies, policies, and other actions that might be used to achieve the priorities ranked with highest importance among the 2 stakeholder groups.

### Data Analysis

The analytical plan will be conducted in 2 steps. First, trained qualitative researchers will conduct open coding of transcripts to identify emergent themes. Members of the research team will read all transcripts and develop an initial list of codes independently. In the open coding process, researchers will identify broad categorical themes that emerge among frequently used words and key phrases in the data. The researchers will then compare emergent codes during regular research meetings to eliminate duplicates and collapse overlapping codes. Discrepancies in coding decisions will be discussed to determine consensus. Upon reaching a consensus, a codebook will be developed, which will be used to purposefully analyze the data. Qualitative analysis will be conducted by independent coders to explore the nuances of the data to develop themes and examine phenomena of interest to generate new concepts [55]. This approach is ideal for studies, such as this one, exploring under-researched areas of inquiry such as local perspectives on GHEs in Kenya [56]. Researchers will independently code approximately 20% of the transcripts and then compare the independently coded data. Interrater reliability will be established by achieving Cohen κ coefficient ≥80%. All qualitative analyses will be conducted using qualitative analysis software (NVivo; QSR International).

## Results

The study activities commenced in late summer 2022, with the recruitment of participants began in August 2022. All 3 phases of this study, including 10 in-depth, one-on-one interviews in phase 1, 2 nominal group activities in phase 2, and 10 in-depth, one-on-one interviews in phase 3 were conducted between September 2022 and February 2023. In each phase, data collection and analysis were conducted concomitantly. The results are expected to be published in 2023.

## Discussion

This qualitative study examines the perceptions of GHEs in relation to the COVID-19 pandemic among Kenyan health care workers and health care administrators working at a facility with a long history of hosting global health activities. It is one of the small but growing number of studies that examine the perceptions of GHEs in LMICs from the local host perspective, and this is the first study to our knowledge to do so in relation to the COVID-19 pandemic. The purpose of this study is to examine participants’ experiences with GHEs in Kenya and the role they are perceived to play in preparing individuals and the health system to address the COVID-19 crisis and their unfolding role in pandemic recovery and its aftermath.

Data collected in this study will be critical to understanding participants’ current and evolving perspectives on GHEs in relation to their work against the backdrop COVID-19 pandemic. Our multiphase study will examine how participants interpret their experiences with GHEs as having advantaged or disadvantaged them and the health system to provide care during a public health crisis, using one-on-one in-depth interviews in phase 1. The second and third phases of the study aim to identify stakeholder priorities and explore recommendations for future GHEs to achieve maximum benefit in the health care system going forward, using NGTs in phase 2, followed by one-on-one in-depth interviews in phase 3, respectively.

Applying a lens of decoloniality to global health research, our study elevates the voices of 2 stakeholder groups that are frequently involved in management and hosting activities associated with GHEs yet are currently underrepresented in global health perceptions literature [11,52]. The findings may reveal areas of strength and potential areas of growth for global health approaches in Kenya and perhaps other LMIC settings. Furthermore, the findings may provide critical data that can inform improved practices to overcome well-documented challenges in global health activities, including issues of equity in global health collaborations as well as sustainability and the effectiveness of programs [20,25].

This study has several strengths and limitations. A key strength of this study is its data collection methodology, which will use highly experienced Kenyan qualitative researchers for data collection to reduce potential bias. Another strength is the inclusion of 2 additional steps in the NGT process. Steps 6 and 7 in phase 2 of this study aim to elicit additional data to garner greater insight into reasoning and decision-making with regard to prioritizing key recommendations to be explored in phase 3. Limitations of this study relate to the contextual aspects of the study setting and its participants, which may limit the generalizability of study findings to other settings. The study site is the flagship hospital of a large, fairly well-resourced network of health facilities, which is not reflective of the wider Kenyan health care system. This could influence the perspectives shared by participants in this study. Likewise, participants will be recruited into 2 broad categories (ie, health care workers and administrators) from among individuals working in disciplines of mental health care, adult medicine, and pediatrics—it is unclear how individuals’ professional views or occupational environments might influence the data. Therefore, the findings from this study may be specific to the local reality and may not be generalizable to other contexts elsewhere in Kenya, sub-Saharan Africa, or other LMICs.

In summary, this exploratory qualitative study will engage 2 key groups of Kenyan stakeholders to gain insight into the role GHEs are perceived to have played in preparing the health care system, and those who work within it, to provide care in an acute public health crisis. Informed by calls to decolonize global health, this study will provide valuable stakeholder-identified priorities and insight into improved practices for the management and accountability of GHEs going forward. The implications of which may provide greater clarity regarding the expected roles and intended purpose of GHEs in Kenya among those who are frequently involved in hosting these activities. A better understanding the role that GHEs play in the professional lives of LMIC stakeholders may shed light on the challenges inherent in hosting GHEs, ultimately informing the development of models for more equitable engagement of LMIC partners.

## Abbreviations

GHE: global health engagement
HIC: high-income country
LMIC: low- and middle-income country
NGT: nominal group technique

## Data Availability

Data sharing is not applicable to this article as no data sets were generated or analyzed during this study.
